# The Role of Dietary Ingredients and Herbs in the Prevention of Non-Communicable Chronic Liver Disease

**DOI:** 10.3390/nu16203505

**Published:** 2024-10-16

**Authors:** Monika Maćków, Tomasz Dziubyna, Tatiana Jamer, Dmytro Slivinskyi, Tomasz Pytrus, Katarzyna Neubauer, Małgorzata Zwolińska-Wcisło, Andrzej Stawarski, Ewa Piotrowska, Dorian Nowacki

**Affiliations:** 1 Department of Human Nutrition, Faculty of Biotechnology and Food Science, Wrocław University of Environmental and Life Sciences, Chełmońskiego 37, 51-630 Wrocław, Poland; monika.mackow@upwr.edu.pl (M.M.); ewa.piotrowska@upwr.edu.pl (E.P.); dorian.nowacki@upwr.edu.pl (D.N.); 2 Regional Specialist Hospital in Wrocław, Research and Development Center, Kamieńskiego 73A, 51-124 Wroclaw, Poland; 3 Unit of Clinical Dietetics, Department of Gastroenterology and Hepatology, Faculty of Medicine, Jagiellonian University Medical College, M. Jakubowskiego 2, 30-688 Kraków, Poland; m.zwolinska-wcislo@uj.edu.pl; 4 2nd Department and Clinic of Paediatrics, Gastroenterology and Nutrition, Wrocław Medical University, M. Curie-Skłodowskiej 50/52, 50-367 Wrocław, Poland; tatiana.jamer@umw.edu.pl (T.J.); tomasz.pytrus@umw.edu.pl (T.P.); andrzej.stawarski@umw.edu.pl (A.S.); 5 Department of Fruit, Vegetable and Plant Nutraceutical Technology, Faculty of Biotechnology and Food Science, Wrocław University of Environmental and Life Sciences, Chełmońskiego 37, 51-630 Wrocław, Poland; dmytro.slivinskyi@upwr.edu.pl; 6 Department and Clinic of Gastroenterology and Hepatology, Wrocław Medical University, Borowska 213, 50-556 Wrocław, Poland; katarzyna.neubauer@umw.edu.pl; 7 Department of Gastroenterology and Hepatology, Faculty of Medicine, Jagiellonian University Medical College, M. Jakubowskiego 2, 30-688 Kraków, Poland

**Keywords:** diet, liver disease, fiber, probiotics, polyphenols, omega-3 fatty acids, herbs

## Abstract

Background: Liver diseases are among the most commonly diagnosed conditions, with the main risk factors being inappropriate lifestyles, including poor diet, excessive alcohol consumption, low physical activity and smoking, including electronic cigarettes. Non-communicable chronic liver diseases also often develop as a result of accompanying overweight and obesity, as well as type 2 diabetes. Methods: The literature on risk factors for non-communicable chronic liver diseases, which show a high strong influence on their occurrence, was analysed. Results: Measures to prevent non-communicable chronic liver disease include the selection of suitable food ingredients that have proven protective effects on the liver. Such ingredients include dietary fibre, probiotics, herbs, various types of polyphenols and fatty acids (omega-3). Conclusions: Because of their liver-protective effects, nutritionists recommend consuming vegetables, fruits, herbs and spices that provide valuable ingredients with anti-inflammatory and anti-cancer effects. These components should be provided with food and, in the case of probiotics, supplementation appears to be important. As a preventive measure, a diet rich in these nutrients is therefore recommended, as well as one that prevents overweight and other diseases that can result in liver disease.

## 1. Introduction

The World Health Organization (WHO) defines non-communicable diseases (NCDs) as long-term chronic illnesses caused by a combination of genetic, physiological, environmental, and behavioral factors [1]. In this article, non-communicable diseases refer to chronic liver disease. Chronic liver disease (CLD), which includes alcoholic liver disease (ALD), metabolic dysfunction-associated steatotic liver disease (MASLD), autoimmune liver disease, and liver cancer, is a major liver disease that is increasingly being diagnosed worldwide [2,3]. Liver diseases cause approximately two million deaths per year, ranking it as the 11th most common cause of death worldwide and accounting for approximately 4% of all deaths overall [4]. It is estimated that CLDs caused around 1.32 million deaths in 2017. Among genders, two-thirds were male and one-third were female [5]. The concept of metabolic dysfunction-associated steatotic liver disease recently emerged, instead of the previously used non-alcoholic fatty liver disease (NAFLD). According to experts, the new definition more accurately reflects current knowledge of hepatic steatosis—a disease associated with complex metabolic dysfunction. According to the definition, MASLD is a steatotic liver disease (SLD) in the presence of one or more cardiometabolic risk factor(s) and the absence of harmful alcohol intake. These cardiometabolic factors include hypertension, elevated levels of plasma triglycerides, low levels of high-density lipoprotein (HDL) cholesterol, dyslipidemia or type 2 diabetes (T2D) mellitus, and overweight or obesity. MASLD is currently the most common chronic liver disease in the world [3]. 

**Figure 1:**
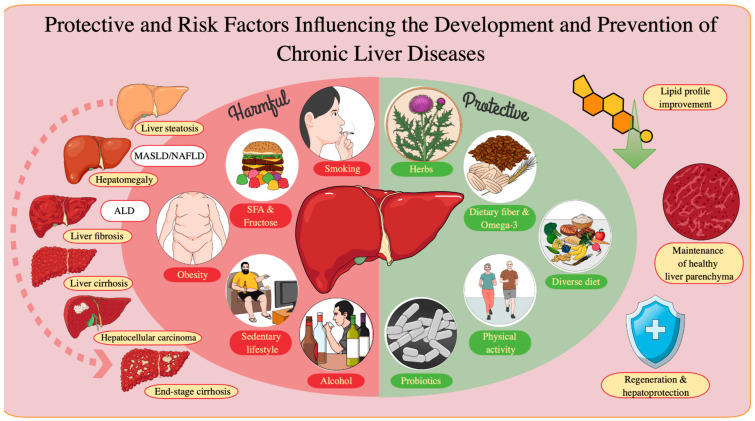
Negative and positive factors influencing the development of non-communicable chronic liver diseases.

A summary of the most important risk factors for non-communicable chronic liver disease is shown in Figure 1.

## 2. Risk Factors of Non-Communicable Chronic Liver Diseases

### 2.1. Cardiometabolic Risk Factors

Risk factors for NCDs are associated with tobacco use, alcohol use, low physical activity, overweight and obesity, food choice, and dental health care [6]. These factors also contribute to the development of liver diseases. Lifestyle factors associated with increased risk of hepatocellular carcinoma (HCC) included past/current smoking, high alcohol consumption, poor diet quality, reduced coffee intake, and prevalent obesity, but not physical activity. The study, conducted on a population living in Hawaii and other regions of the United States, included 753 cases of diagnosed HCC [7]. Factors associated with the occurrence of another liver disease, NAFLD, include high intake of saturated fat and fructose [8], coexistence of central obesity, and insulin resistance, making waist circumference and HOMA index a significant contributor to the occurrence of NAFLD [9]. Changes at the cellular level of adipose tissue increase the production of pro-inflammatory factors (mainly tumor necrosis factor-alpha (TNF-α), interleukin-6 (IL-6), plasminogen activator inhibitor-1 (PAI-1), transforming growth factor beta (TGF-β), C-reactive protein (CRP), enhance the release of significant amounts of free fatty acids, and reduce the secretion of anti-inflammatory factors, such as adiponectin). The consequence of this is the occurrence of increased inflammation that induces the development of MAFLD [10].

The main factors contributing to the development of MASLD include insulin resistance, obesity, or the underlying metabolic syndrome (dyslipidemia, hypertension, and diabetes). Obesity is defined as the excessive accumulation of adipose tissue. Its link with cognitive dysfunction is attributed to the metabolic consequences of visceral adiposity, namely hypertension, dyslipidemia, diabetes type 2, insulin resistance, a constellation referred to as the metabolic syndrome [11,12], and several types of cancer [13]. High BMI increases liver cancer mortality and the incidence of primary liver cancer. Obesity is an independent risk factor for the occurrence and mortality of primary liver cancer [14].

Liver diseases such as MASLD and non-alcoholic steatohepatitis (NASH) are very often causally associated with metabolic disorders such as overweight and obesity, hypertension, dyslipidemia, and diabetes. Obesity is also associated with hemodynamic changes, which may result in changes in the morphology of the heart itself and the functioning of its ventricles. A relationship was identified between the presence and severity of MASLD, in particular NASH and fibrosis, and an increased risk of cardiovascular disease. However, this relationship is dependent on the coexistence of a number of metabolic factors, such as T2D. Importantly, MASLD itself increases the risk of atherosclerosis, cardiomyopathy, arrhythmia, and increases morbidity and mortality from cardiovascular causes [3,15].

### 2.2. Alcohol

According to the WHO reports for 2016, approximately 5.3% of all deaths worldwide were caused by alcohol consumption, which is equivalent to approximately 3 million deaths worldwide [16]. Globally, two-thirds of alcohol-attributable deaths are NCD deaths, and this index is even higher in the European Union (EU), where alcohol consumption is the highest globally and where 75% of all alcohol-attributable deaths are due to NCDs, with cancer (29%) being the leading cause [17]. The causes of chronic liver disease, including liver cancer, vary from region to region, but the most frequent are typically chronic viral hepatitis and alcohol. Alcohol can initiate the development of liver cancer and is related to tumor progression [18]. Drinking more than 60–100 g of alcohol per day contributes to the development of HCC, and alcohol in amounts of 30–50 g/day contributes to the development of liver cirrhosis [19]. Chronic alcohol consumption is a risk factor in 20% to 50% of liver cirrhosis cases worldwide [20]. This is particularly relevant for non-communicable chronic liver diseases such as ALD. It is a liver disorder caused by excessive alcohol consumption. ALD to be one of the important triggers for other liver diseases, including MAFLD. This is because there is a significant infiltration of T lymphocytes in ALD, but their role is not yet well understood. It was suggested that excessive T-lymphocyte production plays an important role in the development of steatohepatitis associated with MASLD [21].

A study of 175 German patients from primary care centers identified, in addition to alcohol, other risk factors for liver fibrosis and cirrhosis, including concomitant obesity and diabetes [22].

### 2.3. Smoking

A WHO report published in 2023 shows that 15 years of the MPOWER program helped reduce global smoking prevalence from 22.8% to 17.0% in 2021 [23]. Smoking a cigarette generates over 4000 chemicals that have a deleterious impact on each part of the human body. It produces three main severe effects on the liver: oncogenic, immunological, and indirect or direct toxic effects. It results in the production of cytotoxic substances, which raises fibrosis and necro-inflammation [24]. Tobacco smoking, as shown by studies and meta-analyses, is significantly associated with NAFLD [25,26,27]. Nicotine, contained in cigarettes, exacerbates hepatic steatosis through increased oxidative stress, hepatic cell apoptosis, and inactivation of adenosine 5-monophosphate-activated protein kinase, leading to increased hepatic lipogenesis. It is also associated with an increase in low-density lipoprotein (LDL) cholesterol, plasma triglycerides and insulin resistance, as well as a decrease in plasma HDL cholesterol levels, which is associated with the occurrence of diabetes, which is also a risk factor for non-infectious liver disease [28]. As one study [29] shows, people diagnosed with MAFLD who also smoke tobacco are also at risk of liver cancer and cardiovascular disease. Smoking is also associated with the incidence of MAFLD among overweight or obese children whose mothers smoked tobacco during pregnancy and were diagnosed with pre-eclampsia and hypertension [30].

In recent years, there was a significant increase in the use of electronic nicotine-dispensing devices, i.e., the so-called e-cigarettes [31]. E-cigarettes gained exceptional popularity, especially among young people, as e-cigarettes are seen as a healthier alternative to smoking classic cigarettes. Within the TackSHS project, a face-to-face survey was conducted in 2017–2018 in 12 European countries (Bulgaria, England, France, Germany, Greece, Ireland, Italy, Latvia, Poland, Portugal, Romania, and Spain). A total of 11,876 participants, representative of the ~15-year-old population in each country, provided information about the electronic cigarette. A total of 65.1% reported using an electronic cigarette in at least one room where smoking was prohibited [32]. A study to determine the effects of e-cigarette flavoring chemicals on HepG2 liver cell toxicity showed that e-cigarette ingredients such as vanillin, ethylvanillin, and ethyl maltol reduce the viability of HepG2 cells caused by long-term exposure to these ingredients [33].

The studies presented here Indicate that it is advisable for pregnant women, those at risk of non-communicable liver disease, or those already diagnosed by a doctor, to stop smoking both traditional cigarettes and e-cigarettes, as smoking is one of the risk factors for liver disease and other associated diseases.

### 2.4. Low Physical Activity

Factors contributing to many chronic diseases include low levels of physical activity. The risk of NAFLD is associated with obesity, insulin resistance, and metabolic syndrome, all of which are associated with low levels of physical activity. As one Korean study showed, prolonged sitting time and reduced levels of physical activity were positively associated with the incidence of NAFLD among middle-aged individuals [34]. Additionally, among the US population, low levels of physical activity increased the risk of MAFLD [35]. However, it should be noted that research on the effects of exercise on chronic liver disease is relatively new [36]. Scientific reports in recent years mainly focused on NAFLD/NASH, MAFLD, and liver cancer. A study of patients diagnosed with liver disease from UK Biobank best demonstrates the benefits of physical activity for this patient group. The study analyzed data from 96,688 participants who recorded their physical activity using a wrist accelerometer. As a result, participants had a reduced risk of both overall CLD and increasing activity by an additional 2500 steps per person per day was associated with a 38% reduction in CLD and a 47% reduction in the development of NAFLD, regardless of the presence of obesity [37].

## 3. Dietary Treatment for the Prevention of Non-Communicable Chronic Liver Disease

Considering the risk factors associated with MAFDL, it seems that the overriding role in the treatment of MAFLD should be attributed to lifestyle modifications aimed at inhibiting the progression of the disease. These changes mainly involve reducing body weight, increasing the level of physical activity, and making a number of dietary changes. A reduction in body weight of as little as 5% significantly improves metabolic parameters and the patient’s clinical condition [38]. Weight reduction improves serum glucose and prevents T2D, improves blood pressure, and reduces triglycerides (TG) and total cholesterol [39,40]. By implementing physical activity and dietary modifications over a period of 0.5–1 years, it is possible to significantly reduce liver enzyme levels, increase cellular sensitivity to insulin, and reduce hepatic steatosis [41].

The literature [42,43,44,45] mentions the Mediterranean diet (MD) among the dietary interventions used in liver diseases. In particular, the capacity of Mediterranean diet to reduce the risk of development and progression of MASLD or NAFLD is attributed to the nutraceutical effect of bioactive compounds and phytochemicals with antioxidant and anti-inflammatory potential such as fibers, monounsaturated and omega-3 fatty acids, and phytosterols [43]. The TANGO study was conducted on a group of Chinese women diagnosed with NAFLD, divided into three groups: one following a Mediterranean diet, another combining the Mediterranean diet with pentadecanoic acid (C15:0) supplementation, and a control group. C15:0, an odd-chain saturated fatty acid found in dairy fat, was linked in epidemiological studies to a reduced risk of metabolic syndrome, T2D, and NAFLD. Elevated plasma levels of C15:0 are associated with lower liver fat content and favorable effects on the gut microbiome. In this study, the Mediterranean diet groups, particularly those with C15:0 supplementation, achieved significantly greater reductions in body weight, liver proton density fat fraction (PDFF), total cholesterol, gamma-glutamyl transferase, and triglycerides compared to the control group [46]. Similar results were obtained in other studies assessing the impact of MD on liver diseases [47,48]. For this reason, the Mediterranean diet is recommended by guidelines on NAFLD published by various associations [3].

## 4. Fiber

Various nutritional societies and bodies promulgated different definitions of dietary fiber (DF) worldwide. The EU regulation on the provision of food information to consumers [49] defines the fiber as “carbohydrate polymers with three or more monomeric units, which are neither digested or absorbed in the human small intestine”. DF was categorized as follows: “edible carbohydrate polymers naturally occurring in the food as consumed, edible carbohydrate polymers obtained from food raw material by physical, enzymatic, or chemical means and which have a beneficial physiological effect demonstrated by generally accepted scientific evidence, and edible synthetic carbohydrate polymers which have a beneficial physiological effect demonstrated by generally accepted scientific evidence”. The most popular definition in nutritional science divides DFs into two subgroups based on their solubility in water: soluble and insoluble [50]. Dietary fiber is found primarily in plant foods, such as whole grains, vegetables, fruits, and legumes [51]. In recent years, a number of scientific reports showed that cellulose plays a significant role in carbohydrate metabolism disorders [52,53], hypertension, and circulatory system diseases [54,55] as well as playing an important role in cancer prevention [56,57,58].

A study of 6613 participants in the United States assessed correlations between fiber intake and the incidence of NAFLD. A reduced risk of NAFLD was found in people who consumed fruits, whole grains, and vegetables which are the source of DF [59]. Other studies also confirm the positive effect of dietary fiber on the occurrence of NAFLD [60,61]. Proper amount of fiber intake, along with a low-energy diet, is linked with weight loss and NAFLD regression [62]. A study conducted in Iran on 158 people with NAFLD showed that fiber, along with other minerals and vitamins, can slow down the NAFLD disease progression [63]. A similar result was obtained by Polish researchers who gave people with NAFLD 28 and 12 g of fiber per day. As a result, a reduction in liver steatosis occurred in both groups, although the effect was similar and more pronounced in patients in the lower fiber group [62]. Other studies also confirmed the effect of fiber on improving liver function in people with NAFLD [64] and reducing the risk of mortality due to NAFLD [65]. Studies show that dietary fiber consumption reduces the risk of MASLD, which is important, especially in people who are at high risk of developing liver disease.

## 5. Polyphenols

Polyphenols are a group of organic compounds naturally found in plants. To date, at least 10,000 substances classified in this group were discovered. Among foods, rich sources of polyphenols include whole grains, spices, vegetables, fruits, chocolate, and beverages such as tea, coffee, and wine [66]. In terms of chemical structure, they are characterized by one or more aromatic rings and one or more attached hydroxyl functional groups [67]. Polyphenols are formed in plants under the action of pathogens or other stress factors, and thus constitute a defense mechanism for plants. This occurs in a similar way in humans, as it was noted that an increase in endogenous antioxidant levels occurs as a result of polyphenols stimulating the antioxidant system, which removes excess free radicals [68,69]. Classification of polyphenols is difficult due to the heterogeneity of chemical structure, but the number of phenolic rings and some structural elements make it possible to divide them into the following groups: flavonoids, phenolic acids, stilbenes, curcuminoids, and lignans [70]. Polyphenols have a variety of biological activities that support human health and can reduce the risk of diseases, including liver disease.

### 5.1. Flavonoids

Flavonoids are a diverse group of polyphenols having a C6-C3-C6 carbon skeleton, which consists of a pyran ring and two aromatic rings. Depending on the degree of oxidation and carbon structure, flavonoids are divided into flavones, flavonols, flavanones, isoflavones, flavanols, and anthocyanins [71]. The most common flavonoids in food are flavonols, soy isoflavones, and flavones. These compounds in foods mainly affect taste, color, protect vitamins and enzymes, and counteract fat oxidation [72].

It seems that the majority of ancient medical therapies that achieved success utilized flavonoids, a practice that persisted to the present day. Quercetin represents the flavonoid subgroup of flavones, demonstrating beneficial pharmacological properties in the management of liver conditions such as liver fibrosis, liver steatosis, fatty hepatitis, and liver cancer. The main portion of quercetin-type flavonols in our diets consists of quercetin glycosides, which are compounds where quercetin is bound to one or two glucose residues or to rutinose. Interestingly, the quantity of quercetin in food can be significantly affected by factors such as growing conditions; for instance, tomatoes grown organically tend to have higher levels of quercetin aglycone compared to conventionally cultivated tomatoes [73]. Overall, fruits and vegetables, especially cherries, cranberries, grapes, blueberries, apples, onions, and peppers serve as the primary sources of naturally occurring dietary quercetin in the typical Western diet [74,75]. Moreover, brewed black tea, along with red table wine and various fruit juices, were also identified as rich dietary sources of quercetin [76,77]. This flavonol is recognized to inhibit the advancement of liver fibrosis by modulating nuclear factor kappa-light-chain-enhancer of activated B cells (NF-κB)-mediated signaling pathways, decreasing the production of cytokines such as TNF-α, IL-6, IL-1β, and IL-8 [78]. Moreover, it boosts antioxidant mechanisms mediated by glutathione and IL-10, while also reducing lipid peroxidation in cases of alcoholic liver disease in rats [79].

Procyanidins, epicatechin, and epigallocatechin and its gallate derivative (EGCG) are the most well-known flavanol molecules. Mostly present in dark chocolate and cocoa, epicatechin was shown to control hepatic and serum lipid profiles by upregulating fatty acid synthase, sterol regulatory element binding protein (SREBP), liver X receptor, and sirtuin (SIRT) [80,81,82]. It was also shown to reduce inflammatory damage and oxidative stress by inhibiting the NF-κB signaling pathway. One of the most often consumed polyphenols in the public is EGCG, which is mostly found in green tea [83]. Numerous in vitro and animal studies provided evidence in favor of the theory that green tea may protect against liver cancer [84,85,86]. Regrettably, there are documented cases of negative effects, primarily hepatitis, from using green tea preparations as an herbal supplement to regulate body weight [87,88]. Green tea’s potential hepatotoxic effects received a lot of study since then. That being said, the daily equivalent of 10.5–32 cups of green tea is equivalent to the amounts of EGCG that may be harmful to humans [89]. Green tea’s protective effects against fatty liver disease are not surprising, as hyperlipidaemia is thought to be a contributing factor and tea polyphenols may increase the liver’s lipid metabolism and lower blood lipid levels [90,91]. It is possible to produce the lipid-lowering effect by preventing lipids from being absorbed through the digestive tract [92]. Moreover, a number of studies demonstrated the protective benefit of tea extracts against cirrhosis and liver fibrosis in rats [93,94]. The study findings show that green tea extract greatly reduced hepatic damage in rats, either through its antioxidant action or by preventing the generation of cytokines caused by lipopolysaccharides (LPS) [95]. However, there is a need for well-designed human clinical trials in this area.

Hesperidin, which belongs to the flavanone group, is found mainly in peppermint and citrus fruits such as grapefruit, lemon, lime, or orange [96,97]. The effectiveness of hesperidin supplementation in the management of NAFLD was assessed in a randomized, double-blind clinical trial. The results indicate that concurrent hesperidin administration and lifestyle modification improves risk factors linked to NAFLD, at least partially, by reducing activation of NF-κB and enhancing lipid profile and insulin sensitivity [98].

### 5.2. Phenolic Acids

This class of polyphenols consists of phenolic compounds, which usually exist in bound form as amides, esters, or glycosides and have one carboxylic group. Coffee and numerous plant-based meals, seeds, fruit skins, and vegetable leaves contain them [99]. Meanwhile, there are three different types of phenolic acids: hydroxycinnamic acids, oleuropeunosides, and hydroxibenzoic acids.

Coffee is a rich source of minerals and antioxidants, phenolic compounds, such as cumaric, chlorogenic, caffeic, and ferulic acids and other bioactive substances that may have positive health effects. Specifically, it was suggested that coffee beans’ other antioxidant compounds, such as chlorogenic acid, may suppress the development of liver cancer [100]. According to the evidence, coffee drinkers had a 40% lower risk of HCC than non-drinkers, and high drinkers have a risk reduction of more than 50% [101]. Coffee drinking is inversely correlated with liver fibrosis, according to a meta-analysis of studies including coffee drinkers and non-drinkers [102]. Based on NAFLD histologic severity, coffee consumption is also inversely correlated with hepatic fibrosis in patients with NAFLD, as well as those with hepatitis C [103,104].

### 5.3. Stilbenes

Stilbenes are plant-based molecules found primarily in red wine, berries, peanuts, and grapes. Some of these compounds are referred to as phytoalexins. Resveratrol is the most researched polyphenol in this group. It can be found in grapes, cocoa, peanuts, mulberries, and soy [105]. Preclinical research identified its protective characteristics on several fronts, including the modulation of oxidative stress and hepatocellular damage to mitigate NAFLD by decreasing pro-inflammatory cytokines and free radicals and boosting the activity of antioxidant enzymes such as cytochrome P450 (CYP) 2E1 and glutathione [106,107]. Resveratrol was shown in numerous studies to have a function in tumor chemoprevention by reducing cancer cell growth and triggering apoptosis in these cells. By delaying the cell cycle and increasing nitric oxide synthase, resveratrol was shown to have anti-proliferative and pro-apoptotic effects on HCC cells [108]. Subsequently, Choi, Chong, and Nam (2009) discovered that resveratrol accumulated cells in the G0/G1 phase and caused dose- and time-dependent liver cancer cell death. Additionally, by inhibiting the antioxidant proteins, resveratrol may make cancer cells more susceptible to oxidative stress [109].

### 5.4. Curcuminoids

One of the main compounds belonging to the curcuminoid subgroup is curcumin. It originates in the rhizomes of the ginger family plant known as turmeric (*Curcuma longa*). Asia is home to naturally occurring turmeric, primarily in India. Because of its strong yellow color, flavor, and aroma, it is frequently used in cooking. Nonetheless, because it contains curcumin, turmeric has been utilized for thousands of years in medicine [110]. There are numerous positive attributes associated with curcumin. Among its additional qualities include anti-inflammatory, antioxidant, and anti-cancer properties [111]. According to recent research, curcumin and its derivatives greatly lower the metabolism of fat and glucose in the liver and serum, which inhibits inflammatory markers in both organs. Through a number of metabolic pathways, including the peroxidase proliferator-activated receptor (PPAR) signaling system and the adenosine 5′-monophosphate-activated protein kinase (AMPK) signaling network, it also controls the metabolic illness known as NAFLD [112,113,114]. Furthermore, curcumin activates PPAR-γ, which inhibits hepatic steatosis and hepatic stellate cell activation [115]. A meta-analysis shows that curcumin supplementation alone, in combination with physical activity, dietary guidance, and/or lifestyle modifications, can lower blood levels of total cholesterol, total triglycerides, alanine aminotransferase, aspartate aminotransferase, fasting blood insulin, LDL, and as well as homeostasis model assessment of insulin resistance and waist circumference. Nonetheless, this resulted from the application of doses of curcumin ranging from 80 to 1500 mg and extra advice. So far, research is yet to be able to clearly determine the appropriate dose of curcumin to use alone or in combination with other forms of physical activity, dietary advice, or other lifestyle modifications [116].

### 5.5. Lignans

A large class of naturally occurring substances known as lignans are produced via the shikimic acid biosynthesis pathway [117]. The structural scaffold of lignans is comprised of two or more phenylpropanoid units [118]. The monomers that constitute lignans are propenyl benzene, allyl benzene, cinnamyl alcohol, and cinnamic acid. The richest sources of lignans are sesame seeds and linseed. In addition, lignans are also found in soy, oats, wheat, fish, meat, wine, coffee, and tea [119]. Studies on rats showed that linseed, which contains a molecule called diglucoside, reduces lipid peroxidation in the liver, the accumulation of lipids in the liver and serum cholesterol [120]. So far, clinical studies are yet to be conducted to evaluate the effect of lignans on the prevention of liver diseases.

## 6. Herbal Approaches in Liver Disease Prevention

Herbs are usually available as dietary supplements and are often not reliably assessed for safety and effectiveness. Herbal products can protect the liver from liver damage, but their effects must be documented in randomized clinical trials to allow for the rational use of these agents. Improper use can contribute to liver damage, which significantly contributes to serious health consequences.

### 6.1. Milk Thistle

Milk thistle (*Silybum marianum*) has been used in the treatment of liver diseases in traditional medicine for over 2000 years. The standardized extract of milk thistle (silymarin) contains flavonolignans (silybin A, silybin B, isosilybin A, isosilybin B, silychristin, and silydianin), fatty acids, and polyphenolic compounds. These components have synergistic positive effects on the metabolism of the human body, particularly the liver function [121,122]. The most biologically active compound in milk thistle is silybin, which is mainly found in the seeds and fruits of the plant [122]. Silybin exhibits antioxidant activity, protecting hepatocytes from damage caused by free radicals and lipid peroxidation. Additionally, it has antifibrotic effects by inhibiting the activation of hepatic stellate cells, thereby reducing the fibrosis. Furthermore, silybin acts as a barrier against toxins, preventing them from entering liver cells. Due to these properties, milk thistle has significant potential in the prevention and treatment of liver diseases [122]. Moreover, when silymarin was administered for four weeks to diet-induced obese mice, improvements were observed in intrahepatic lipid accumulation, plasma triglyceride, and levels of HDL and LDL [123]. According to Song et al. in model mice with ALD, silymarin (200 mg/kg) was shown to reduce oxidative stress. This was achieved by giving ethanol at a dosage of 5 g/kg body weight every 12 h for a total of three doses. Moreover, silymarin therapy stopped reductions in glutathione and lipid peroxidation, as well as elevations in TNF-α and alanine aminotransferase levels [124]. For many years, silymarin has been utilized as a hepatoprotective substance in basic research, and as additional studies demonstrate the substance’s significance in a variety of liver illnesses, this use of the substance is only expected to grow. Treatment with silymarin is not commonly utilized, however, as it requires evidence from well-organized trials and standardization of the techniques for assessing therapeutic success, particularly in the promising case of NAFLD [125].

### 6.2. Greater Burdock

Greater burdock (*Arctium lappa*), similar to milk thistle, belongs to the Asteraceae family. It is a primary component of the KIOM2012H herbal formula due to its wide range of healthy effects, including antioxidant, anticancer, and anti-inflammatory effects. These properties are related to the presence of the lignan arctigenin. Greater burdock exhibits significant hepatoprotective effect, protecting the liver from damage caused by lead and cadmium. This mechanism involves the reduction in oxidative stress and the inhibition of inflammatory processes in the liver. The activation of signaling pathways such as Akt/GSK-3β (protein kinase B/glycogen synthase kinase 3 beta) and the increased activity of antioxidant enzymes such as SOD (superoxide dismutase) and catalase further enhance the protective effects of this herb on liver tissue [126]. In vitro and in vivo studies show the potential effect of the KIOM2012H herbal formula in the prevention and treatment of NAFLD [127]. It is important to emphasize that this formula also includes Glycyrrhiza uralensis, Magnolia officinalis, and Zingiber officinale, which are yet to be the subjects of clinical trials. However, there is evidence based on in vitro and in vivo studies.

### 6.3. Ginger

Ginger (*Zingiber officinale*), similar to turmeric, has long been used in traditional medicine and cuisine. Extracts from ginger and its biologically active components, especially gingerol and shogaol, exhibit antioxidant, anticancer, anti-inflammatory, and lipid-lowering effects, which translate into hepatoprotective benefits [128]. Ginger is one of the four components of the KIOM2012H herbal formula with potential hepatoprotective effects by improvement in serum lipid levels, including cholesterol. Furthermore, ginger influences the regulation of LDL oxidation processes [127]. Numerous in vitro and in vivo studies indicate the hepatoprotective potential of ginger by induction of apoptosis, inhibition of the liver cancer cell cycle, reduction in the migration and growth of cancer cells, and the showing of antioxidant effects on hepatocytes [129]. However, there is a lack of clinical studies on the above effects, which seems to be necessary for a complete understanding of ginger’s therapeutic potential in the context of liver disease treatment and prevention.

### 6.4. Glycyrrhiza Uralensis and Magnolia Officinalis

Glycyrrhiza uralensis and Magnolia officinalis are other components of the KIOM2012H herbal formula. They show potential in the prevention and treatment of liver diseases through antioxidant activity and modulation of key signaling pathways associated with lipogenesis and inflammation in the liver [127]. In the case of Glycyrrhiza uralensis, its positive effects are primarily attributed to three compounds: formononetin, 3′-methoxyglabridin, and glabridin. Network pharmacology and molecular docking studies identified connections between the active ingredients of Glycyrrhiza and their molecular targets in the body, as well as assessed the stability of bonds between these ingredients and target proteins. These studies suggest that Glycyrrhiza uralensis affects key signaling pathways, such as mitogen-activated protein kinase 3 (MAPK3), signal transducer and activator of transcription 3 (STAT3), and AKT1. The impact on these pathways suggests a role for Glycyrrhiza in the prevention and treatment of alcohol-induced liver damage [130]. However, there are no clinical studies confirming this effect.

Despite the promising results of preclinical studies on Magnolia officinalis, there is a clear lack of adequate research on its therapeutic potential. Most available studies focus on mechanisms of action at the cellular and molecular levels. However, comprehensive analyses confirming the effectiveness of this plant in a clinical context are lacking. Therefore, further clinical research is necessary to confirm the therapeutic potential of Magnolia officinalis in liver diseases.

### 6.5. Traditional Chinese Medicine

Traditional Chinese medicine uses a variety of herbs to improve patients’ health. In a retrospective cohort study conducted on a population of diabetic patients in Taiwan (*n* = 81,105) aimed at assessing the effect of integrated treatment—Chinese and Western medicine—on the risk of HCC, it was shown that the use of herbal preparations reduces the risk of HCC by 41%. Among the most commonly recommended herbs in traditional Chinese medicine are Salvia miltiorrhiza, Astragalus membranaceus, Pueraria lobata, Rheum palmatum, and Dendrobium nobile. Their effects are based on antioxidant mechanisms that effectively neutralize free radicals, protecting liver cells from oxidative stress. For example, Salvia miltiorrhiza demonstrates the ability to modulate signaling pathways, reducing the levels of pro-inflammatory cytokines such as TNF-α. Astragalus membranaceus, in turn, not only has anti-inflammatory effects, but also supports liver cell regeneration. Pueraria lobata regulates lipid metabolism and improves the level of cholesterol and TG. Rheum palmatum improves liver regeneration. Dendrobium nobile shows the ability to inhibit the proliferation of cancer cells, making it a promising agent in the prevention of HCC [131].

**Figure 2:**
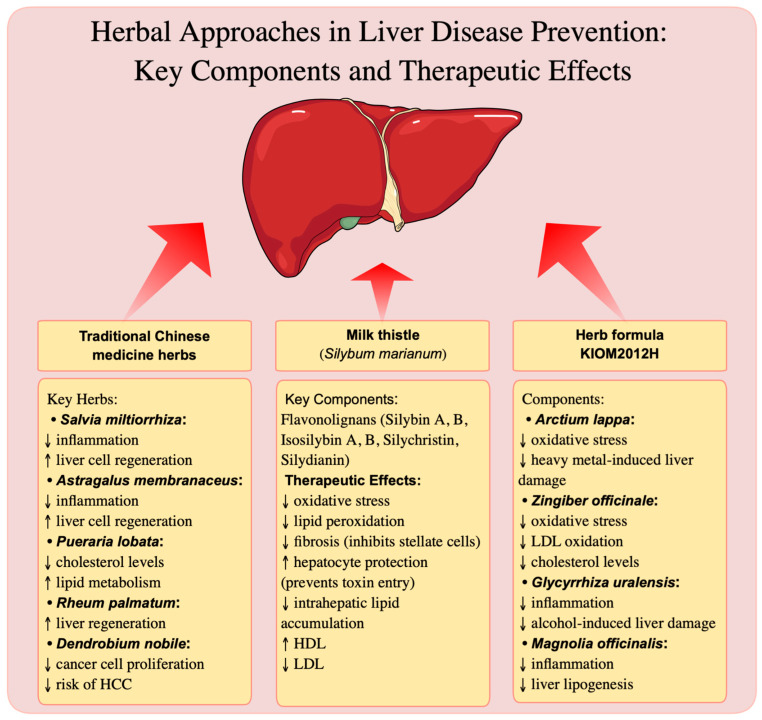
Schematic representation of key herbal components in liver disease prevention. Signs: ↑ increasees, ↓ decreases.

The key herbal components that were discussed in this subchapter are summarized in Figure 2. The current knowledge regarding the potential of herbs in liver disease prevention is largely based on preclinical studies, while clinical studies are scarce. Herbs certainly have potential applications for patients at risk of liver diseases, but they should be used with great caution. For example, the prolonged chewing of betel nuts (*Areca catechu* L.), which were used for centuries in traditional medicine, is linked to the development of liver cirrhosis and HCC. The main active compound, arecoline, exhibits genotoxic, hepatotoxic, and carcinogenic effects, raising concerns about the safety of long-term consumption of these nuts. Epidemiological studies indicate a significantly higher risk of liver disease development in individuals who regularly chew betel nuts, regardless of the presence of other risk factors, such as hepatitis B or C virus infection [129]. Therefore, extensive randomized clinical trials are necessary to accurately assess the efficacy of herbs, their active ingredients, and extracts in the treatment and prevention of liver diseases. Clinical studies are essential not only in the context of the therapeutic potential of herbs, but also to determine therapeutic doses, potentially toxic doses, to identify interactions with other medications, and any possible side effects.

## 7. Unsaturated Fatty Acids and Phospholipids in Liver Disease Prevention

The primary sources of *n*-3 (omega-3) and *n*-6 (omega-6) polyunsaturated fatty acids in the human diet are fatty marine fish, nuts, and vegetable oils. These products also provide fat-soluble vitamins, which have a beneficial impact on metabolic health. Among these vitamins, vitamins D and E are particularly important in the context of health and the main sources are fish as well as nuts that were mentioned earlier [132].

Fatty acids from the *n*-3 group, such as eicosapentaenoic acid (EPA) and docosahexaenoic acid (DHA), play a crucial role in preventing NAFLD. Their beneficial effect is related to improvement in lipid metabolism and reduction in liver inflammation. EPA and DHA are preferentially oxidized by hepatocytes, which limits their accumulation in the form of TG and reduces the risk of liver steatosis. Additionally, *n*-3 polyunsaturated fatty acids (PUFAs) have the ability to inhibit the expression of genes involved in the de novo lipogenesis (DNL), thereby lowering TG synthesis in the liver. The anti-inflammatory activity of *n*-3 PUFAs are also playing a significant role by reducing the level of pro-inflammatory cytokines and decreasing oxidative stress in liver cells, thereby preventing the progression of NAFLD to more progressed form, such as NASH [133]. An important hepatoprotective mechanism of omega-3 fatty acids is their ability to modulate the ratio of omega-6 to omega-3 fatty acids in the diet. A high omega-6 to omega-3 ratio exhibits pro-inflammatory effects, which seems to be significant in the context of NAFLD, where chronic inflammation is a key risk factor. A randomized, controlled, double-blind clinical trial involving 176 participants (*n* = 176) demonstrated that omega-3 supplementation leads to a reduction in this ratio, contributing to decreased liver inflammation and improved lipid metabolism. A crucial role here is played by omega-3’s influence on activity of peroxisome proliferator-activated receptor alpha (PPARα) and sterol regulatory element-binding proteins (SREBP-1c), which regulate the expression of genes involved in lipogenesis and fatty acid oxidation. PPARα stimulates fatty acid beta-oxidation, leading to their oxidation instead of accumulation as TG, while omega-3 inhibition of SREBP-1c decreases lipid synthesis in the liver. Additionally, omega-3 improves hepatic insulin sensitivity by improving insulin signaling, which is a key factor of NAFLD prevention and treatment, as insulin resistance is one of the main factors in the development of this disease. Improved insulin sensitivity reduces lipogenesis and supports processes associated with fatty acid oxidation, which translates into a reduction in liver steatosis [134].

Another significant aspect of the hepatoprotective action of omega-3 fatty acids is their impact on metabolism related to fat accumulation and the risk of developing liver diseases. Omega-3 fatty acids, especially EPA and DHA, effectively lower the fatty liver index (FLI), lipid accumulation product (LAP), and visceral adiposity index (VAI). These effects are strongly associated with liver fat content and insulin resistance, which are key elements in the pathophysiology of NAFLD. In a randomized, controlled clinical trial involving 60 patients with T2D and NAFLD, supplementation with 2000 mg of omega-3 for 12 weeks led to significant improvement in FLI, LAP, and VAI compared to the placebo group. The reduction in these factors indicates decreased fat accumulation in the liver and improved overall lipid metabolism [135].

Nuts, such as almonds, walnuts, pistachios, and hazelnuts, exhibit hepatoprotective effects, as proved by numerous epidemiological and clinical studies. A meta-analysis of 12 studies involving over 90,000 participants (*n* = 92,621) found that regular nut consumption is associated with a significant reduction in the risk of developing NAFLD. This effect is primarily attributed to the high content of monounsaturated and polyunsaturated fatty acids (MUFA and PUFA) and antioxidants such as vitamin E and polyphenols. Bioactive substances in nuts, such as ellagic acid and alpha-linolenic acid (ALA), play a key role in modulation of the body’s inflammatory response. Nut consumption can lead to a significant reduction in inflammatory markers, such as TNF-α. Another preventive factor in the context of liver diseases is the ability of nuts to support weight control by improving satiety and reducing visceral fat accumulation [136].

In a randomized, controlled study involving 68 participants, it was shown that regular consumption of cashew nuts and cashew nut oil can lead to a significant reduction in cardiovascular risk factors, including reduction in LDL cholesterol, inflammatory markers such as apolipoprotein B (apo B), gamma-glutamyltransferase (GGT), and liver enzymes: aspartate aminotransferase (AST) and alanine aminotransferase (ALT). Additionally, the study found that cashew consumption reduces neck circumference, which is an important indicator of visceral fat reduction. Similar to other nuts, cashews contain high amounts of MUFA, including oleic acid, which is shown to have anti-atherosclerotic activity [137].

Despite the numerous health benefits of nut consumption, there is a risk of contamination with aflatoxins—toxins produced by molds of the Aspergillus. In the context of NAFLD, where the risk of disease progression is increased, it is important to choose nuts from controlled sources to minimize exposure to these toxins. Nonetheless, nuts remain an important dietary component that can support the prevention and treatment of liver diseases when the source and quality is appropriate [138].

Fish oil, rich in omega-3 fatty acids, supports lipid metabolism in the liver, reducing TG accumulation by stimulating beta-oxidation. Additionally, fish oil has anti-inflammatory and antioxidant effects, helping to reduce oxidative stress induced by alcohol and supporting the integrity of the intestinal barrier, protecting against endotoxemia [139]. Such hepatoprotective effects in the context of alcohol suggest a significant potential for fish oil in protecting the liver from other toxic factors. However, further research is needed.

Another important component with hepatoprotective potential is phospholipids, especially polyenylphosphatidylcholine (PPC) derived from soybeans. Phospholipids are fundamental components of cell membranes, which not only support the structure and function of hepatocytes, but also participate in the regeneration of these cells. Studies indicate that PPC supplementation improves liver function, which is evidenced by reduced levels of aminotransferases (ALT and AST) and improved lipid metabolism. Phospholipids also show antioxidant and anti-inflammatory activity that reduces the progression of liver disease, including fibrosis. Long-term clinical studies on PPC showed that its use may significantly improve the health of patients with NAFLD and be useful as a supportive therapy [140].

One of the potent sources of omega-3 fatty acids and phospholipids is krill oil. While fish oil contains omega-3 fatty acids in the form of triglycerides, krill oil has them bound to phospholipids, specifically phosphatidylcholine, which improves their absorption and bioavailability. This means that lower doses of krill oil can provide similar or even superior benefits compared to higher doses of fish oil. Additionally, krill oil contains natural antioxidants such as astaxanthin, offering stronger anti-inflammatory and antioxidant effects than fish oil [141]. These factors make krill oil a more efficient option for managing conditions such as NAFLD by reducing triglycerides and improving liver fat metabolism.

Euphausia pacifica, also known as North Pacific krill, similarly to Euphausia superba (*Antarctic krill*), produces oil (*E. pacifica* oil) that is rich in omega-3 fatty acids, but with a higher concentration of phospholipids and a unique compound—8-hydroxyeicosapentaenoic acid (8-HEPE). 8-HEPE demonstrates potent physiological effects, including activating peroxisome proliferator-activated receptors (PPARs), which play a key role in lipid metabolism. This makes *E. pacifica* oil particularly effective in reducing triglyceride accumulation, improving lipid profiles, and mitigating hepatic steatosis [142].

Krill oil is not only beneficial for its high bioavailability and antioxidant properties, but also offers a more sustainable alternative to traditional fish oil sources. The krill fishery, particularly in the Antarctic, is subject to strict environmental regulations that ensure sustainable harvesting practices. With large krill populations and precautionary catch limits, this resource remains abundant and less vulnerable to overfishing. Moreover, advancements in extraction techniques further reduced the environmental footprint of krill oil production, making it a responsible choice for those seeking both health benefits and eco-conscious options [143].

Diet plays a fundamental role in hepatoprotection, as the appropriate selection of nutrients can significantly influence the course and progression of liver diseases. The Mediterranean diet, rich in products that are good sources of unsaturated fatty acids, polyphenols, and vitamins, is an example of a dietary pattern with high potential for modulating metabolic processes in the liver. Additionally, this diet has an anti-inflammatory property, which is an important factor in the pathogenesis of liver diseases [144]. The proper selection of products and dietary patterns can positively impact liver health indicators, making nutrition one of the key elements of hepatoprotection.

## 8. Probiotics and Microbiota

The gut microbiota can play a crucial role in the development and prevention of various liver diseases. Dysbiosis, or an imbalance in the gut microbiome, can contribute to liver disorders through increased intestinal permeability, chronic inflammation, and metabolic changes [145]. Metabolic dysfunction-associated steatotic liver disease, alcoholic liver disease, and hepatocellular carcinoma are associated with alterations in gut microbiota [146]. Endotoxins (LPS, which are a component of the outer membrane of Gram-negative bacteria) are released into the circulation after bacterial lysis and may reach the liver via the portal vein. LPS can activate complement, the kinin system, leukocytes, platelets, and endothelial cells and increase the synthesis of pro-inflammatory cytokines, inducing oxidative stress contributing to the inflammatory process in MASLD [147,148].

Probiotics can modulate intestinal microbiota, improve intestinal barrier function, and exert immunomodulatory effects, potentially preventing harmful bacteria and their products from entering the circulation and causing liver damage [148,149]. Studies showed that probiotic use can lead to improvements in liver function tests, decreased inflammatory markers, and reduced blood cholesterol levels [148].

It seems important to modify the gastrointestinal microbiota by consuming probiotic bacteria naturally occurring in food or contained in dietary supplements to reduce the progressive inflammation in the liver by modulating the gut microbiome, reducing inflammation, and enhancing intestinal barrier function [148,150].

A randomized study by Alisi et.al to demonstrate the effect of a 4-month supplementation with a composition of 8 probiotic strains (*Bifidobacterium breve*, *Bifidobacterium longum*, *Bifidobacterium infantis*, *Lactobacillus acidophilus*, *Lactobacillus plantarum*, *Lactobacillus paracasei*, *Lactobacillus delbrueckii* subsp. *bulgaricus*, and *Streptococcus thermophilus*) in obese children with steatohepatitis showed that the intake of this composition by the study group had a positive effect on the course of the disease compared with placebo. A significant decrease in liver enzymes: ASP and ALT were observed, along with a reduction in the degree of hepatic steatosis in the study group of subjects [151].

A further double-blind study by Duseja et al. [152] also showed a beneficial effect of a probiotic on the course of steatohepatitis. In this study, patients treated for 12 months with lifestyle modification and a multi-strain probiotic (*Lactobacillus paracasei* DSM 24733, *Lactobacillus plantarum* DSM 24730, *Lactobacillus acidophilus* DSM 24735 and *Lactobacillus delbrueckii* ssp. *bulgaricus* DSM 24734, *Bifidobacterium longum* DSM 24736, *Bifidobacterium infantis* DSM 24737, *Bifidobacterium breve* DSM 24732, and *Streptococcus thermophilus* DSM 24731) were observed to reduce inflammation, significantly improve liver histology, as well as reduce alanine transaminase (ALT) activity and improve the profile of pro-inflammatory cytokines. Probiotics administered in MAFLD modify the composition of the intestinal microbiota, improve intestinal barrier function, and the metabolism of lipid compounds and reduce pro-inflammatory cytokines. They may therefore be a beneficial management strategy in patients with fatty liver disease. The literature data do not clearly clarify the long-term effects of probiotics in this group of patients. There is a need for further research in this area, especially in view of the growing global epidemic of MAFLD.

A systematic review and meta-analysis was recently published that also provides convincing evidence that probiotics are beneficial in treating the complication of MAFLD, liver cirrhosis, demonstrating reversal of hepatic encephalopathy, potential improvement in liver function, improvement in quality of life, and regulation of gut dysbiosis. Furthermore, the apparent safety profile suggests that probiotics are a promising intervention in the treatment of liver cirrhosis [153].

While lifestyle modifications remain the primary intervention for NAFLD, probiotics offer a promising adjunct therapy due to their ability to positively influence gut microbiota composition and potentially mitigate liver disease progression [154]. Understanding the complex interactions between the gut microbiota and liver diseases may lead to novel prevention and treatment strategies for these disorders.

## 9. Conclusions

Non-communicable chronic liver disease is one of the most frequently diagnosed diseases that is a consequence of an inappropriate lifestyle, including poor nutrition, smoking, and alcohol consumption together with inadequate physical activity.

The dietary model associated with the world’s most common chronic liver disease, MASLD, is the Western diet. Leading features of this diet are low fruit and vegetable content, a high proportion of animal products, simple sugars, and processed foods. In contrast, the diet recommended for patients diagnosed with MASLD is the Mediterranean diet. The components of this diet, whose sources are primarily fruits and vegetables, are in turn important in the prevention of MASLD. Their preventive effect is usually related to the prevention of cardiometabolic risk factors, which in turn are associated with MASLD.

For instance, the protective effect of dietary fiber on non-communicable chronic liver disease was proven, and its consumption may prevent excessive body weight and MASLD. Dietary fiber can also contribute to beneficial gut microbiota composition, which, together with probiotic supplementation, may in the future also become one of the methods for the prevention of non-communicable liver diseases. Numerous scientific studies observed positive results associated with the use of polyphenols in the prevention of MASLD or liver cancer, such as catechins, silymarin, resveratrol, anthocyanins, and flavonoids. These different types of polyphenols are present in coffee, fruits, and vegetables, among others, and should be part of a balanced diet due to their anti-inflammatory and anti-cancer effects. It is worth noting that coffee already has a place in recommendations for MASLD patients. Fatty acids, especially EPA and DHA, regulate pathways involved in fat metabolism, regulate fatty acid metabolism in tissues, and improve the blood lipid profile. The supply of these fatty acids from fish, plant products, or in the form of supplementation should therefore be recommended to people who are at high risk of non-infectious liver disease. Additionally, the constituents of certain herbs influence liver metabolism and the prevention of various diseases. However, it should be borne in mind that such ingredients should only be used under medical supervision, as inappropriate use, particularly in the form of dietary supplements, can cause liver injury and have other serious health consequences.

In conclusion, a proper diet, rich in products of plant origin, and thus active ingredients that show a protective effect against chronic non-infectious liver diseases, not smoking and not drinking alcohol, along with adequate physical activity, are crucial in the prevention of chronic liver diseases.
